# Epidemiological characteristics and influencing factors of rehabilitation length of stay among hospitalized patients after knee arthroplasty in Beijing, China: a retrospective study

**DOI:** 10.3389/fepid.2026.1919766

**Published:** 2026-10-29

**Authors:** Jun Liu, Yi Wang, Shasha Jin, Jiangen He

**Affiliations:** 1 Department of Sports Rehabilitation, Beijing Xiaotangshan Hospital, Beijing, China; 2 Office of Hospital Administration, Beijing Jishuitan Hospital, Capital Medical University, Beijing, China

**Keywords:** China, epidemiology, knee arthroplasty, length of stay, rehabilitation

## Abstract

**Study design:**

Retrospective epidemiological study.

**Objectives:**

To analyze the epidemiological characteristics of patients receiving inpatient rehabilitation after knee arthroplasty, evaluate their rehabilitation LoS, and explore the factors influencing rehabilitation LoS.

**Methods:**

This retrospective study consecutively enrolled patients receiving inpatient rehabilitation after primary total or unicompartmental knee arthroplasty at a tertiary rehabilitation hospital in Beijing from 2023 to 2026. Data on demographics, insurance type, comorbidities, length of stay, Modified Barthel Index and hospitalization costs were collected. Statistical analyses included the Mann–Whitney U test, Spearman's rank correlation analysis, and a Gamma generalized linear model to explore predictors of rehabilitation length of stay.

**Results:**

Among all included admissions, there were 60 admissions (58.3%) covered by Beijing local medical insurance and 43 admissions (41.7%) with off-site medical insurance. The median length of rehabilitation stay was 25 days, and the median time to rehab admission from primary surgery to admission to the rehabilitation center was 5 days. The median total rehabilitation hospitalization cost was 28,337.1 Chinese Yuan (3,987.1 US dollars), among which rehabilitation training expenses accounted for 49.23%. After adjusting for confounding factors, only type of medical insurance was identified as an independent influencing factor of length of hospital stay (*P* < 0.05).

**Conclusions:**

This study implies that orthopedic hierarchical care and integrated rehabilitation models may help optimize rehabilitation length of stay and ease patients’ economic burden. Further investigation of cross-institutional rapid referral pathways is needed.

## Introduction

Knee osteoarthritis is a prevalent chronic degenerative joint disease among middle-aged and elderly populations, which commonly leads to persistent joint pain and limited joint mobility. With population aging, the number of elderly patients suffering from knee disorders keeps increasing. As a primary therapeutic strategy for severe knee osteoarthritis, knee arthroplasty can effectively relieve joint pain and restore knee function. Furthermore, it brings multiple long-term additional benefits, including helping prolong patients’ life expectancy (1).Over the past two decades, the volume of knee arthroplasty procedures has been rising due to population aging and the growing prevalence of overweight and obesity in Western societies (2).A similar trend has also emerged in China. Taking Beijing as an example, the degree of population aging is continuously deepening. By the end of 2023, the registered population aged 60 years and above in the city reached 4.316 million, accounting for 30.2% of the total registered population, marking the first time this proportion exceeded 30% (3).

To curb the irrational growth of medical expenditures and build a high-efficiency medical insurance payment system, the National Healthcare Security Administration (NHSA) of China launched the Diagnosis-Related Groups (DRG) payment model in 2019 (3). The core objectives of DRG payment are to conserve medical insurance funds, optimize the allocation of medical resources, alleviate patients’ financial burden, and promote the balanced advancement of cost control and healthcare quality (4). However, this policy has not yet been implemented in rehabilitation medicine for conditions such as stroke, spinal cord injury, and orthopedic disorders. In China, domestic studies investigating the length of hospital stay and overall medical expenditure related to knee arthroplasty remain relatively scarce.

Against this backdrop, this retrospective study analyzed and summarized the clinical data of patients receiving inpatient rehabilitation after knee arthroplasty who were treated at the Orthopedic Rehabilitation Center of a tertiary rehabilitation hospital in Beijing over the past four years. This study intends to establish baseline data on the clinical characteristics of inpatient rehabilitation following knee arthroplasty in Beijing, fill the regional gap in targeted large-sample relevant data, deliver precise references for clinical rehabilitation diagnosis and treatment, and provide evidence-based evidence for optimizing inpatient management, rationally controlling medical insurance expenditures.

## Methods

### Study design and individuals

Patients who received primary total knee arthroplasty (TKA) or unicompartmental knee arthroplasty (UKA) and were subsequently transferred to the Department of Rehabilitation Medicine (an Inpatient Rehabilitation Facility, IRF) of a tertiary rehabilitation hospital in Beijing for inpatient rehabilitation between September 2023 and April 2026 were enrolled as research subjects.

### Discharge criteria

The length of rehabilitation stay before patients were discharged home was jointly determined by attending physicians, rehabilitation therapists, patients and their family members. No patients were forced to discharge early unilaterally by the medical institution. Actual discharge criteria are influenced by both clinical and social factors. Clinical stability is required for discharge. Discharge clinical stability was assessed by attending surgeons using consistent criteria: stable vital signs, absence of surgical site infection and major postoperative complications, adequate pain control with oral analgesics, and the ability to complete basic transfer and mobilization. Some patients set return to home as their discharge goal, while others aim for community reintegration.

### Inclusion criteria

(1) Age ≥ 18 years old; (2) Diagnosed with knee osteoarthritis and underwent unilateral primary TKA or UKA; (3) Complete clinical records, detailed cost statements and outcome indicators.

### Exclusion criteria

Cases with revision arthroplasty, infectious arthritis, bone tumors, fractures or other conditions other than primary osteoarthritis; (2) Patients transferred to other hospitals for further rehabilitation during hospitalization.

This was a retrospective observational study, and all eligible patients were consecutively recruited within a fixed study period. All regression analyses in this study were regarded as exploratory. A total of 109 hospitalization records were initially screened. Four were excluded due to transfer to another hospital during hospitalization, and two were excluded because they were admitted for knee revision surgery.

### Sensitivity analysis

The primary statistical analysis included 103 admissions from 98 patients. Five patients received staged bilateral knee arthroplasty and had two separate admissions for left and right knee replacement, respectively. To eliminate the potential influence of within-patient correlation and test the robustness of the findings, a sensitivity analysis was conducted by enrolling only the first admission of each patient (*n* = 98). Both univariate analyses and generalized linear models were repeated with the same variable configuration and model settings as the primary analysis.

### Data collection

The following data were extracted from the hospital electronic medical record system.

Gender, age, type of medical insurance, surgical type, major comorbidities and complications (urinary tract infection, deep Vein Thrombosis, osteoporosis, anemia, hypoalbuminemia, type 2 diabetes mellitus, hypertension, coronary heart disease, intracerebral hemorrhage,cerebral infarction, hyperlipidemia, heart failure, etc.). Date of surgery, time to rehab admission, total length of hospital stay, surgical type, and activities of daily living (ADL) scores at rehabilitation admission and discharge.

### Outcome assessment

The Shah version of the Modified Barthel Index (MBI) was used to evaluate basic activities of daily living. The scale consists of 10 items with a total score ranging from 0 to 100. Each item is divided into five functional grades, and higher scores indicate better independence in activities of daily living. Assessments were performed at the bedside on the day of admission and the day of discharge by rehabilitation physicians with standardized training in scale administration.

### Rehabilitation intervention

All patients received one-on-one rehabilitation during hospitalization. Physical therapy was delivered 10 times per week, with each session lasting 40–60 min. The main contents included knee range-of-motion training, lower-limb muscle strength training, transfer and gait training, balance training, and activities of daily living guidance. Cryotherapy, ultrasound, transcutaneous electrical nerve stimulation (TENS) and acupuncture were selectively applied based on postoperative swelling and pain status as well as patients’ willingness, and these interventions were not mandatory. The costs of physical agent modalities and acupuncture were excluded from rehabilitation training fees but were included in the total hospitalization cost.

### Hospitalization cost data

Total hospitalization costs included rehabilitation therapy fees (only functional rehabilitation training; physical agent modalities, acupuncture and other traditional Chinese medicine-related treatments were excluded), physical-agent-modality therapy fees, traditional Chinese medicine therapy fees (including acupuncture and other TCM treatments), pharmaceutical expenses (western and traditional Chinese medicines), laboratory-examination fees, consumable-material fees, nursing fees, ward-bed fees, and medical consultation fees. Caregiver-service fees were not included.

### RMB-to-USD conversion method

All medical costs were originally recorded in Chinese Yuan (RMB), with data collected from September 2023 to April 2026. Segmented conversions from RMB to US Dollars (USD) were performed using corresponding official monthly/annual average exchange rates during the study period:

Costs incurred in 2023: divided by 7.0467 to convert to USD
Costs incurred in 2024: divided by 7.1217 to convert to USD
Costs incurred in 2025: divided by 7.1429 to convert to USD
Costs incurred from January to April 2026: divided by 6.92995 to convert to USD (5)

### Statistical analysis

Excel 2019 was used for double-data entry by two independent researchers to establish the database, and SPSS 21.0 software was adopted for statistical analyses. For descriptive statistics, the normality of LoS was assessed using the K-S (Lilliefors) test and Q–Q plots. Q–Q plots showed that length of stay (LoS) followed a non-normal distribution, and the K-S (Lilliefors) test yielded *P* < 0.05.

### Grouping for Mann-Whitney U

Subjects were grouped according to the research objectives as follows: (1) By gender: male grou*p* = 1, female grou*p* = 2; (2) By comorbidities and complications: presence = 1, absence = 0; (3) By medical insurance type: Beijing local medical insurance = 1, off-site medical insurance = 2.

### Correlation analysis

Mann–Whitney U was applied to compare the differences in LoS among groups for categorical baseline variables. Spearman's rank correlation analysis was performed to examine correlations between non-normally distributed continuous variables and LoS. A generalized linear model (GLM) with Gamma distribution and log link function was used to identify independent predictors of rehabilitation length of stay. Variables with *P* < 0.2 in univariate analyses were included in the multivariable model. For categorical variables, off-site medical insurance was set as the reference category for insurance type, female for gender, presence of osteoporosis for osteoporosis status, and presence of coronary heart disease for coronary heart disease status. Robust standard errors were adopted for the model. The Akaike information criterion (AIC) was used to evaluate model fit. Model diagnostics were performed using Pearson residuals and Q-Q plots, and no obvious violation of model assumptions was observed. Regression coefficients (*β*), exponentiated coefficients [exp(*β*)], and their corresponding 95% confidence intervals (95% CIs) are presented in Table 1.

**Table 1:** Results of generalized linear model analysis for independent predictors of length of hospital stay.

Parameter estimation										
Parameter	*β*	Std. Error	95% Wald confidence Time to rehab admission		Hypothesis testing			Exp (β)	95% Wald confidence intervals for Exp(β)	
			Lower limit	Upper limit	Wald *χ*^2^	df	*P* value		Lower limit	Upper limit
（Intercept）	3.458	0.3390	2.794	4.123	104.068	1	< 0.001	31.761	16.343	61.723
Male	0.187	0.1658	−0.138	0.512	1.274	1	0.259	1.206	0.871	1.669
Female	0a	.	.	.	.	.	.	1	.	.
Local	0.290	0.1230	0.049	0.531	5.545	1	0.019	1.336	1.050	1.700
Off-site	0a	.	.	.	.	.	.	1	.	.
Absence of osteoporosis	−0.166	0.1377	−0.436	0.104	1.455	1	0.228	0.847	0.647	1.109
Presence of osteoporosis	0a	.	.	.	.	.	.	1	.	.
Absence of coronary heart disease	0.314	0.2028	−0.083	0.712	2.404	1	0.121	1.370	0.920	2.038
Presence of coronary heart disease	0a	.	.	.	.	.	.	1	.	.
ADL score at admission	−0.009	0.0054	−0.020	0.002	2.625	1	0.105	0.991	0.981	1.002
Time to rehab admission	−0.007	0.0049	−0.017	0.002	2.387	1	0.122	0.993	0.983	1.002
(scale mark)	0.349b									

Dependent variable: Length of hospital stay. Model: (Intercept), type of medical insurance,osteoporosis, Time to rehab admission, admission ADL, Gender, Coronary heart disease. This parameter is redundant and therefore set to zero.

### Ethical approval

This study was approved by the Ethics Committee of Beijing Xiaotangshan Hospital, with the ethical approval number [2026] Ethics Approval No. (24). All patients signed informed consent forms upon admission.

## Results

### Sensitivity analysis

After restricting the dataset to one admission per patient (*n* = 98), the univariate Mann–Whitney U test, spearman's rank correlation analysis, and generalized linear model analyses were repeated. Sensitivity analysis restricted to 98 unique patients yielded consistent findings for insurance type (*β*=0.302, Exp(*β*) = 1.353, 95%CI: 1.061–1.725, *P* = 0.015), which was compatible with the primary analysis. No substantial changes in effect size or statistical inference were observed, which verified the statistical robustness of our results.

### Individual characteristics

The analysis of this study revealed 14 male admissions (13.6%) and 89 female admissions (86.4%). The age range of participants was wide, from a minimum of 54 years to a maximum of 81 years, with a mean age of 68.96 ± 5.44 years and a median age of 69 years. There were 60 admissions (58.3%) covered by Beijing local medical insurance and 43 admissions (41.7%) with off-site medical insurance. The mean length of rehabilitation stay was 28.57 ± 18.63 days, with a median of 25 days. Among all admissions, 85 cases (82.5%) received total knee arthroplasty (TKA), and 18 cases (17.5%) received unicompartmental knee arthroplasty (UKA).

### ADL scores

The modified Barthel Index (Shah version) at hospital admission was 56.52 ± 13.7, with a median value of 55.

The modified Barthel Index (Shah version) at hospital discharge was 77.01 ± 12.3, with a median value of 77.

### Time from surgery to rehabilitation admission

The time to rehab admission from knee arthroplasty to admission to the rehabilitation center averaged 7.67 ± 11.0119 days, with a median of 5 days and a range of 0 to 69 days (Table 2).

**Table 2:** Distribution of time from surgery to rehabilitation admission.

Days	Patients
0 2	2 9
3	16
4 5 6 7 8 9 10 11–15 17–19 26–28 35–36 52–69	24 19 11 4 2 2 1 4 2 2 2 3

### Hospitalization costs

The mean total rehabilitation hospitalization cost was 33,084.2 ± 21,037.4 Chinese Yuan (4,658.3 ± 2,949.9 US dollars), and the median total cost was 28,337.1 Chinese Yuan (3,987.1 US dollars). Rehabilitation training expenses accounted for 49.23% of the total cost.

### Univariate analysis

Spearman's rank correlation analysis indicated that hospitalization costs, admission ADL score, and waiting time to rehab admission were significantly correlated with total LoS(*P* < 0.05) (Table 3). Mann–Whitney U demonstrated statistically significant differences in LoS between patients with and without osteoporosis(*P* < 0.05) (Table 4).

**Table 3:** Effects of continuous variables on LOS in patients after knee arthroplasty.

Outcome	Statistic	Hospitalization costs	Age	ADL score at admission	Time to rehab admission	ADL score at discharge
LOS	Correlation coefficient	0.876	0.103	−0.260**	−0.212*	0.029
	Sig.	< 0.001	0.303	0.008	0.032	0.771
	N	103	103	103	103	103

**Table 4:** Effects of categorical variables on LOS Among patients after knee arthroplasty.

Variables	P
Gender Type of surgery	0.118 0.534
Type of medical insurance	0.092
Urinary tract infection Deep Vein Thrombosis Osteoporosis Anemia Hypoalbuminemia Type 2 diabetes mellitus Hypertension Coronary heart disease Intracerebral hemorrhage Cerebral infarction Hyperlipidemia Heart failure	0.331 0.924 0.005 0.229 0.506 0.461 0.496 0.124 0.272 0.834 0.361 0.774

### Gamma generalized linear model analysis

Spearman's rank correlation analysis revealed a significant positive correlation between length of stay and total hospitalization cost (*P* < 0.05), indicating that longer hospital stay was associated with higher total hospitalization cost. Since total hospitalization cost was a secondary outcome resulting from length of stay, it was not incorporated into the generalized linear model with length of stay as the dependent variable. Variables with *P* < 0.2 in Mann–Whitney U and Spearman's rank correlation analysis were incorporated into the Gamma Generalized Linear Model. After adjusting for confounding factors, only type of medical insurance was identified as an independent influencing factor of LoS (*P* < 0.05) (Table 1).

## Discussion

Three key findings emerged from this study. First, Given their large proportion, patients with non-local medical insurance form a non-negligible population, patients covered by non-local medical insurance have significantly shorter hospital stays than those with local medical insurance. Second, home rehabilitation is associated with some risks, and poor recovery can occur in a minority of patients. Third, lower ADL scores and shorter time to rehab admission were not independent predictors of longer rehabilitation length of stay. Patients who were readmitted due to poor outcomes after home-based rehabilitation did not exhibit longer lengths of hospital stay.

Previous studies on KA have mostly identified age and comorbidities as major influencing factors (6). However, our cohort consisted of patients transferred to the rehabilitation ward after the acute postoperative phase. The effects of conventional clinical factors were attenuated, while social factors became prominent. Variations in discharge timing between insurance subgroups demonstrate heterogeneous discharge goals in KA rehabilitation patients, indicating the need for tailored management strategies for the two populations. In fact, the benefits of inpatient rehabilitation are not as obvious as presumed. Multiple studies from Europe and America have reported no significant differences in pain severity, functional recovery and patient satisfaction between home-based and inpatient rehabilitation, and thus recommended home rehabilitation protocols with superior cost-effectiveness (7–9). An Australian study demonstrated that inpatient rehabilitation could not improve patients’ physical function at 26 weeks postoperatively compared with supervised home rehabilitation involving 2–3 group outpatient rehabilitation sessions (10). Another Australian study provided robust evidence that one-on-one individual outpatient therapy yielded no better short- or long-term outcomes after TKA than group outpatient rehabilitation or supervised home rehabilitation with two scheduled one-on-one outpatient visits. Individual therapy providers deliver treatment at higher costs without generating substantial clinical improvements (11). Thus, full-course inpatient rehabilitation may not be necessary. For patients with local medical insurance, novel rehabilitation models integrating inpatient, outpatient, community and home-based rehabilitation should be actively promoted, guiding patients covered by local medical insurance to complete most rehabilitation care in non-inpatient settings. Second, we observed shorter LOS in patients with non-local insurance. This finding may be related to unmeasured social factors, yet further research is required to verify this hypothesis. After surgery, these patients usually only have access to inpatient rehabilitation in Beijing. Their discharge criterion is the ability to travel by plane or train, rather than the capacity for long-distance walking with satisfactory stability and coordination. Furthermore, trans-provincial medical insurance is commonly associated with practical constraints such as reduced reimbursement rates. Therefore, once patients achieve clinical stability and can walk short distances, both clinicians and patients generally opt for early discharge, and patients return to their hometowns to complete subsequent rehabilitation at home. It is recommended to further promote the development of regional orthopedic medical centers led by the National Orthopedic Center of China (12), and use medical insurance policies to further encourage patients to receive total knee arthroplasty in nearby medical institutions (13, 14).

Lower ADL scores and shorter time to rehab admission were not independent predictors of longer rehabilitation length of stay. A US study reported that patients entered rehabilitation at an average of 4 days after joint arthroplasty (15). In Beijing's local care pathway where patients are transferred to rehabilitation facilities immediately after surgery, the timing of rehabilitation intervention is comparable to that of developed nations. Rapid rehabilitation referral channels may improve medical service efficiency without prolonging length of stay. Further research is needed to verify this hypothesis.

Home-based rehabilitation carries inherent risks. Some patients receiving home rehabilitation were readmitted for inpatient rehabilitation up to 69 days after surgery due to unsatisfactory recovery of knee range of motion and self-care ability. A study by Showalter demonstrated that patients encountered obstacles in home rehabilitation, including persistent pain induced by post-discharge rehabilitation exercises and failure to translate rehabilitation knowledge and skills acquired in hospital into daily home practice. In addition, a considerable proportion of patients suffered from prolonged pain and persistent limitations in knee joint function (16–18). However, statistical analyses suggested that this did not appear to prolong their hospital stay compared with patients admitted for rehabilitation immediately after surgery. These findings indicate that for patients with unsatisfactory recovery after home-based rehabilitation, inpatient rehabilitation can provide timely remediation of functional impairments without increasing their time and economic costs, which helps relieve concerns among patients undergoing home rehabilitation.

This single-center retrospective real-world study has several limitations. First, Potential selection bias exists in this study. Given the variations in clinical practices and charging standards across medical institutions in different regions, caution should be exercised when generalizing the study findings. Second, rehabilitation protocols and discharge criteria were not standardized under routine real-world clinical practice. Knee-specific metrics such as KOOS, WOMAC were unavailable, and the Modified Barthel Index only captures global functional independence rather than knee-specific recovery. Third, Medical prices remained stable during the study period, but price fluctuations may still exert an influence on the study results.

Furthermore, this study only collected data during inpatient rehabilitation without long-term post-discharge follow-up, making it impossible to evaluate the impacts of relevant variables on patients’ full-cycle medical costs and long-term functional recovery. In China, health economic research on rehabilitation following knee arthroplasty remains scarce. This single-center retrospective real-world study provides a foundation for subsequent longitudinal and interventional studies on post-KA rehabilitation, facilitating the translational application of academic findings into clinical practice.

Future research directions include expanding the sample size to conduct multi-center studies. In addition, research by Chan Zeng suggested potential racial disparities in rehabilitation outcomes following knee arthroplasty (19). Given the scarcity of relevant domestic studies in China, further research is required to verify whether the comparable efficacy of inpatient and home rehabilitation observed in Western populations can be generalized to Chinese patients.

## Conclusions

Improving China's orthopedic hierarchical diagnosis and treatment system centered on the National Orthopedic Center of China and establishing a mature inpatient-outpatient-community-home integrated rehabilitation model may help shorten rehabilitation length of stay for patients undergoing knee arthroplasty and potentially reduce their economic burden. Rapid referral pathways linking surgical hospitals and rehabilitation institutions hold promise for optimizing care; further development and evaluation of such pathways are warranted.

## Data Availability

The raw data supporting the conclusions of this article will be made available by the authors, without undue reservation.
